# Maternal autoimmune antibodies alter the dendritic arbor and spine numbers in the infragranular layers of the cortex

**DOI:** 10.1371/journal.pone.0183443

**Published:** 2017-08-18

**Authors:** Jeanelle Ariza, Jesus Hurtado, Haille Rogers, Raymond Ikeda, Michael Dill, Craig Steward, Donnay Creary, Judy Van de Water, Verónica Martínez-Cerdeño

**Affiliations:** 1 Department of Pathology and Laboratory Medicine, Sacramento, CA, United States of America; 2 Institute for Pediatric Regenerative Medicine and Shriners Hospitals for Children Northern California, Sacramento, CA, United States of America; 3 MIND Institute, Sacramento, CA, United States of America; 4 Department of Rheumatology/Allergy and Clinical Immunology, UC Davis, Davis, United States of America; Universidade Federal do ABC, BRAZIL

## Abstract

An association between maternal IgG antibodies reactive against proteins in fetal brain and an outcome of autism in the child has been identified. Using a mouse model of prenatal intraventricular administration of autism-specific maternal IgG, we demonstrated that these antibodies produce behavioral alterations similar to those in children with ASD. We previously demonstrated that these antibodies bind to radial glial stem cells (RG) and observed an increase in the number of divisions of translocating RG in the developing cortex. We also showed an alteration in brain size and as well as a generalized increased of neuronal volume in adult mice. Here, we used our intraventricular mouse model of antibody administration, followed by Golgi and Neurolucida analysis to demonstrate that during midstages of neurogenesis these maternal autism-specific antibodies produced a consistent decrease in the number of spines in the infragranular layers in the adult cortical areas analyzed. Specifically, in the frontal cortex basal dendrites of layer V neurons were decreased in length and volume, and both the total number of spines—mature and immature—and the spine density were lower than in the control neurons from the same region. Further, in the occipital cortex layer VI neurons presented with a decrease in the total number of spines and in the spine density in the apical dendrite, as well as decrease in the number of mature spines in the apical and basal dendrites. Interestingly, the time of exposure to these antibodies (E14.5) coincides with the generation of pyramidal neurons in layer V in the frontal cortex and in layer VI in the occipital cortex, following the normal rostro-caudal pattern of cortical cell generation. We recently demonstrated that one of the primary antigens recognized by these antibodies corresponds to stress-induced phosphoprotein 1 (STIP1). Here we hypothesize that the reduction in the access of newborn cells to STIP1 in the developing cortex may be responsible for the reduced dendritic arborization and number of spines we noted in the adult cortex.

## Introduction

Autism spectrum disorders (ASD) are defined by a pattern of qualitative abnormalities in reciprocal social interaction, communication, and repetitive interests and behaviors. Recent estimates indicate that 1 in 68 children in the United States are impacted by ASD [1]. Identified regions of the brain that are affected in ASD include the cerebral cortex and the cerebellum [2, 3]. Neuropathological changes in affected brain areas include alterations in cell numbers and connectivity [4–6]. These alterations are produced by prenatal and postnatal changes in the normal patterns of cortical development, including stem cell function and neurite outgrowth [7].

Maternal IgG isotype antibodies cross the placenta beginning by week 17 of gestation to equip the immunologically naïve fetus with a subset of antibodies that provide protection against a myriad of possible infectious agents [8]. These maternal IgG antibodies are known to persist for up to 10 weeks after birth [9]. However, together with immunoprotective IgG antibodies, autoantibodies that react to fetal ‘self’-proteins can also cross the placenta. Gestational transfer of maternal autoantibodies with reactivity to fetal proteins is an established cause of congenital abnormalities in the context of maternal autoimmune disorders. Therefore, brain-reactive antibodies have the potential to exert substantial effects on the fetal brain through their interaction with target antigens. This interaction can take several forms including receptor activation, receptor blockade, and/or reducing the effective level of a soluble protein.

Recently, a strong association between maternal IgG antibodies reactive against proteins in fetal brain (MAU^ab^) and an outcome of autism in the child, has been independently identified by several groups [10, 11]. A particular pattern of reactivity to fetal brain proteins at approximately 37 and 73 kDa was initially observed uniquely among mothers of children with ASD [12]. This same pattern of reactivity was observed in prospectively collected mid-gestation blood samples from mothers who went on to have a child with autism [13], supporting the possibility that these antibodies may directly affect neurodevelopment *in utero*. We have previously demonstrated that maternal autoantibodies are reactive to the identical proteins in both human and mouse fetal brain. Our more recent studies demonstrate, for the first time, that lactate dehydrogenase A and B (LDH), stress-induced phosphoprotein 1 (STIP1), and collapsin response mediator protein 1 (CRMP1) comprise the primary antigens corresponding to the previously described 37 and 73 kDa (doublet) proteins recognized by MAU^ab^ [14]. Further, ASD children from mothers with specific reactivity to LDH, STIP1 and CRMP1 have elevated stereotypical behaviors compared to ASD children from mothers lacking these antibodies[15].

Using a mouse model of prenatal intraventricular administration of MAU^ab^, we have demonstrated that these produce behavioral alterations similar to those in human autism [16, 17]. We have also demonstrated a strong link between maternal autoantibodies and fetal brain development, since these antibodies bind to radial glial (RG) stem cells, the primary neural stem cell in most regions of the developing brain. Consequently, we observed an increase in the number of divisions of translocating RG cells in the subventricular zone of the developing cortex. Based on these data, we proposed that maternal autoantibodies are reducing the length of the neurogenic period in the cerebral cortex and ganglionic eminence. In addition, we showed an alteration in brain size and weight in the adult mice prenatally exposed to these autoantibodies, as well as a generalized increased of neuronal volume. We hypothesized that the alteration in brain and neuronal volume could be due to several factors such as an alteration of dendritic arbors and/or an accumulation of products of cellular degeneration [16].

Dendrites and spines are the main neuronal structures receiving input from other neurons and glial cells, and their number and morphology is one of the crucial factors determining how signals coming from individual synapses are integrated. Dendrites contain dendritic spines that are microscopic membrane protrusions comprising the receptive postsynaptic compartment of excitatory synapses in the brain [18, 19]. Spine morphology, number and density are crucial factor determining the strength and stability of the synaptic transmission[20–24]. Dendrites of a single neuron can contain hundreds to thousands of spines and a typical mature spine has a single synapse located at its head. Dendrite and spine modifications have been described in many disease states, however much remains to be understood about the characteristics of neuronal dendrites and dendritic spines in autism and related disorders. Based on its morphology, dendritic spines have been classified as thin, stubby, and mushroom, among others [25]. Spines are classified into specific morphologies based on the spine's head to neck diameter ratio. For example, mushrooms spines have a large head and a narrow neck, stubby spines have no obvious constriction between the head and the attachment to the shaft, and thin spines have a smaller head and a narrow neck [26]. Spine morphology is related with function. Mushrooms and stubby spines are considered mature spines because they are stable, persist for long periods of time, and form strong excitatory synapses [27, 28], while thin spines are considered immature because there are highly motile, unstable, and often short-lived, representing weak or silent synapses [29]. Filopodia are long and thin protrusions that are precursors to spines.

Currently much remains to be learned about the characteristics of neuronal dendrites in individuals with autism. The data collected from human tissue is scarce but suggest that an alteration in dendritic and spine density is related to the pathogenesis of autism [30–34]. Williams et al. specifically quantified spines in the human cerebral cortex. However, this study was limited to 4 cases of autism with mental retardation and only two cases presented with qualitatively decreased spine densities. In addition, one of the cases suffered from uncontrolled seizures [32]. On the other hand, Hutsler et al. identified spine density increases in apical and basal dendrites of pyramidal neurons located in the in the superficial layers of the frontal, temporal and parietal lobes, and in the apical dendrites of layer V in the temporal lobe. Not all the 10 ASD cases presented with this increase in spine density, but high spine densities were associated with decreased brain weights and were most commonly found in ASD subjects with lower levels of cognitive functioning [34]. Spine number and size may be dependent on the area of cortex, the cortical layer, and the age of the individual examined, among other variables. It is also possible that since autism is a very heterogeneous syndrome, different individuals present with different dendrite and spine modifications. Data collected from autism-like mouse models also present with an alteration in dendritic and spine density and may reflect an impairment in the developmental organizational processes of synapse stabilization and elimination, or pruning [30].

Here, we investigated the presence of a potential alteration in the dendritic arbor and spine population in adult mice prenatally exposed to autism-specific maternal autoantibodies. We utilized our intraventricular model of maternal autoantibody administration [16, 17] and, using the Golgi method and Neurolucida, determined the morphology of the dendritic arbor including the number and type of dendritic spines in each layer of the cerebral cortex of treated animals.

## Materials and methods

We randomly assigned pregnant dams to MAU^ab^ or MTD^ab^ administration on embryonic day 14. Pups were born, and when adult (8 weeks of age, n = 5 per group) were perfused, their brains were extracted and Golgi staining was performed. Once Golgi was finalized and 100 μm sections cut, we selected a pyramidal neuron within each of the supragranular (II-III) and infragranular layers (IV-V) in both prefrontal and occipital cortices from each animal. In total 80 neurons were analyzed. Using Neurolucida we traced the pyramidal neuron’s body, dendrites and spines. Each dendrite was traced using a different color. Spines were classified in thin and mature spines. Thin spines included long spines with no head including thin spines and filopodia. Mature spines included spines with stubby and mushroom morphology. We analyzed data obtained for apical and basal dendrites separately. Specifically, we quantified the number of basal dendrites, the total length and volume of apical and basal dendrites, the number of dendrite terminations and bifurcations of apical and basal dendrites, as well the number of type-specific spines.

### Plasma samples

For this study, maternal samples were obtained from the CHARGE Study at the University of California, Davis M.I.N.D. Institute. To be eligible for study participation, the children of the maternal samples used were between the ages of 24 and 60 months at the time of enrollment, and the maternal postnatal sample was acquired at this time. This study protocol followed the ethical guidelines of the most recent Declaration of Helsinki and was approved by the institutional review boards at the University of California, Davis, and the State of California Office of Human Research Protection. Informed consent of the mothers was obtained prior to participation in the study. All study participants received a subject identification number and all personal identifiers were removed from the sample prior to use in the study. Following characterization of the maternal autoantibody profile by fetal brain western blot, IgG was purified from plasma under sterile conditions using Protein A/G columns and dialyzed against sterile saline. Prepared IgG samples were tested for bacterial contamination using an LPS-detecting turbidity assay, and only samples verified to be sterile and pyogen free were used. The experimental group included embryos injected with IgG obtained from plasma from a mother of a child with autism who had a high titer of IgG reactivity to the 37- kDa (lactate dehydrogenase or LDH) and 73-kDa (stress-induced phosphoprotein 1 or STIP1, and collapsin response mediator protein 1 or CRMP1) fetal brain proteins (MAU^ab^), that was used in our previous studies [6, 17]. In the control group embryos were injected with IgG isolated from plasma from a mother of a typically developing child who did not have fetal brain-reactive IgG (MTD^ab^). Embryos in two pregnant animals were injected per group.

### Animals

The UC Davis Institutional Animal Care and Use Committee approved all experiments included here. Animals were housed in cages containing 4 mice with food and water *ad libitum*. Food, water, temperature, humidity, ventilation, and light cycle were check and husbandry records collected daily. We carefully observed animals for changes in health status, including for pain and distress. Signal of pain and distress included change in body weight, external physical appearance, clinical signs (e.g., inability to reach food and water, lethargy or decreased mental alertness, labored breathing, inability to remain upright), and/or significant changes in behavior. No mice presented with conditions that resulted in significant pain that could not be alleviated by analgesics. Mice were euthanized using a lethal dose of anesthetic (4% isoflurane) and perfused using intracardiac perfusion of saline solution. Pregnant adult female Swiss Webster mice were purchased from Charles River. We used female Swiss Webster because, based in our previous experience [35–37], embryos from this strain are easier to inject *in utero* and the survival rate is higher than that in other mice strains. All animals were housed at the University of California, Davis animal facilities. The number of animals used in each experiment was minimized.

### Antibody administration

Pregnant dams were randomly assigned to injection of MAU^ab^ or to injection of MTD^ab^. Timed pregnant mice were anesthetized on embryonic day (E)14, an abdominal incision made through the skin and the abdominal muscular layer, and temporarily exposed the uterine horns. 0.5–1.0 μL containing 10 μg of purified MAU^ab^ or MTD^ab^ was then injected directly into the cerebral ventricle of each embryo by passing a 33-gauge micropipette through the uterine wall and into the cerebral ventricle. All pups were injected in each pregnant dam. The uterine horns were replaced, and the muscular layer and skin sutured closed.

### Golgi

**Fig 1:**
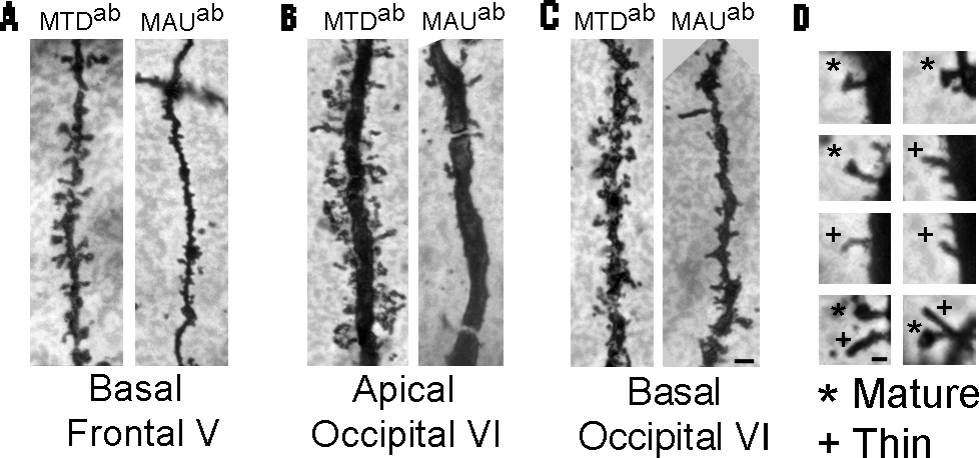
Golgi-stained dendrites. A. Basal dendrites in frontal cortex layer V of MTD^ab^ and MAU^ab^ treated mice. B. Apical dendrites in occipital cortex layer VI of MTD^ab^ and MAU^ab^ treated mice. C. Basal dendrites in occipital cortex layer VI of MTD^ab^ and MAU^ab^ treated mice. D. Representative morphology of spines classified as mature (*) and thin (+). Scale bar: A-C: 2.5 μm; D. 0.25 μm.

We post-fixed tissue blocks encompassing layer I to VI with PFA and 1.5% picric acid in 0.1 M PBS for 24h, rinsed them with PBS, transferred them to a 0.02% osmium tetroxide solution in PBS for 10–30 min under gentle shaking in the dark, rinsed again, and immersed them in 3% potassium dichromate in deionized water in the dark at 4°C for 24h. We cut the whole cortex in 100 μm sections and gently placed them in the impregnation solution of 1.5% silver nitrate at room temperature (RT) in the dark for 24 h. After that period, we rinsed and mounted sections on gelatin-coated slides, dried slices at RT and dehydrated in ascending series of ethanol, cleared in xylene, mounted with non-acidic synthetic balsam, and dried at RT for 24h[38, 39], Fig 1.

### Neurolucida

**Fig 2:**
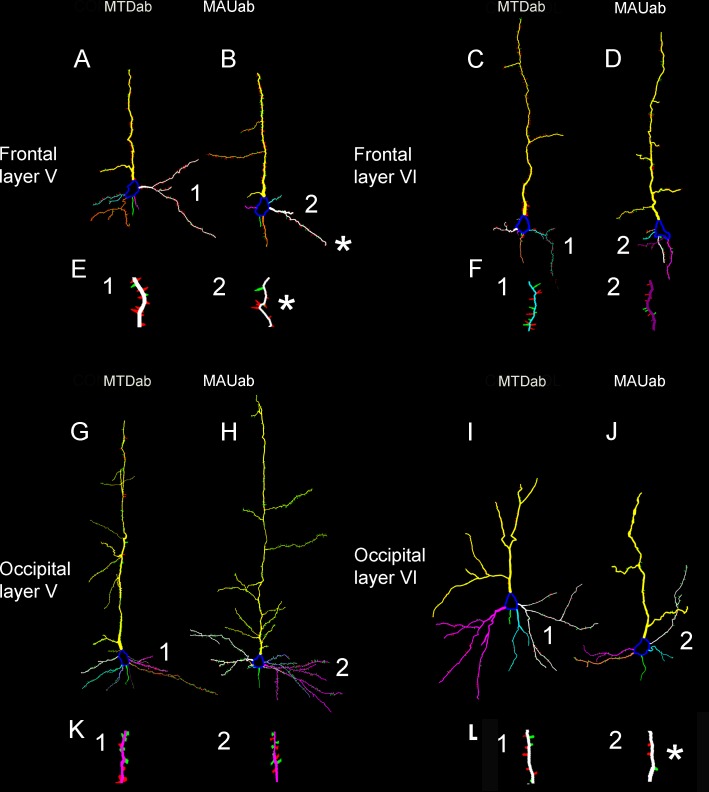
Neurolucida reconstruction of representative pyramidal neurons in infragranular layers of the cortex of MTD^ab^ and MAU^ab^ treated mice. A-F: Frontal cortex. G-L: Occipital cortex. Somata are in blue, apical dendrites in yellow, axonal initial segments in green, and basal dendrites in other colors (tail, orange, pink, white, purple). Neurons in A,B,G,H belong to layer V, while those in C,D, I, J belong to layer VI. E, F, K, L are higher magnification images of basal dendrites located above—indicated with numbers (1 and 2)—demonstrating spine reconstructions. Mature spines are in red, while immature spines are colored in green. Asterisks indicate basal dendrites with a significant decrease in mature spines (E2 and L2).

We selected six pyramidal neurons in the frontal cortex and six pyramidal neurons in the occipital cortex of each mouse brain, one neuron per layer. Researcher was blinded to diagnosis. We considered as frontal cortex the 3 most rostral sections in the frontal pole and occipital cortex the 3 most caudal sections located in the occipital pole. We identified pyramidal neurons by their characteristic triangular soma and apical dendrites extending toward the pial surface. Neuron were chosen based on their presence of a complete Golgi impregnation of dendrites and spines with a limited amount of staining artifacts. We scanned each neuron under 60x oil immersion magnification by varying the depth of the Z plane. We generated bidimensional reconstructions of the three-dimensional dendritic tree including spines for each neuron, Fig 2. We used Neurolucida 7.5 software (MBF Bioscience) and an Olympus microscope equipped with a high-resolution digital camera, a mechanical stage, and an x-y-z axis encoder.

### Statistics

The number of dendrites and spines in MAU^ab^ and MTD^ab^ cases was compared using T-test. A p value of 0.05 was used for statistical significance.

## Results

The dendritic arbor of pyramidal neurons in all layers of the prefrontal cortex (MTD^ab^ and MAU^ab^) was in general smaller in length and volume than those in the occipital cortex (Fig 1). Pyramidal cells in each layer presented with similar dendritic length and volume, except for those in layer VI that were noticeable smaller than in the other layers. We detected a difference in length and volume of basal dendrites in layer V of the prefrontal cortex between the MAU^ab^ group and the MTD^ab^ group. We did not find any change in the length and volume of apical and basal dendrites in the rest of the layers, nor in the number of basal dendrites, the number of dendrite terminations, or the number of bifurcations between the MAU^ab^ and the MTD^ab^ groups.

The type of predominant spine type in both the MAU^ab^ and the MTD^ab^ groups were mature spines (stubby and mushroom) while thin spines were lower in number in both apical and basal dendrites (Fig 2). An exception to this was found in layer V of the occipital cortex where thin spines outnumbered mature spines. A very small number of branched spines were also present in most of the neurons. We did not find any change in the number or type of spines in layers II and III in throughout the cortex, however we found a decrease in the number of specific spine types in the infragranular layers of the cortex when compared the MAU^ab^ and MTD^ab^ groups.

### Frontal cortex

**Fig 3:**
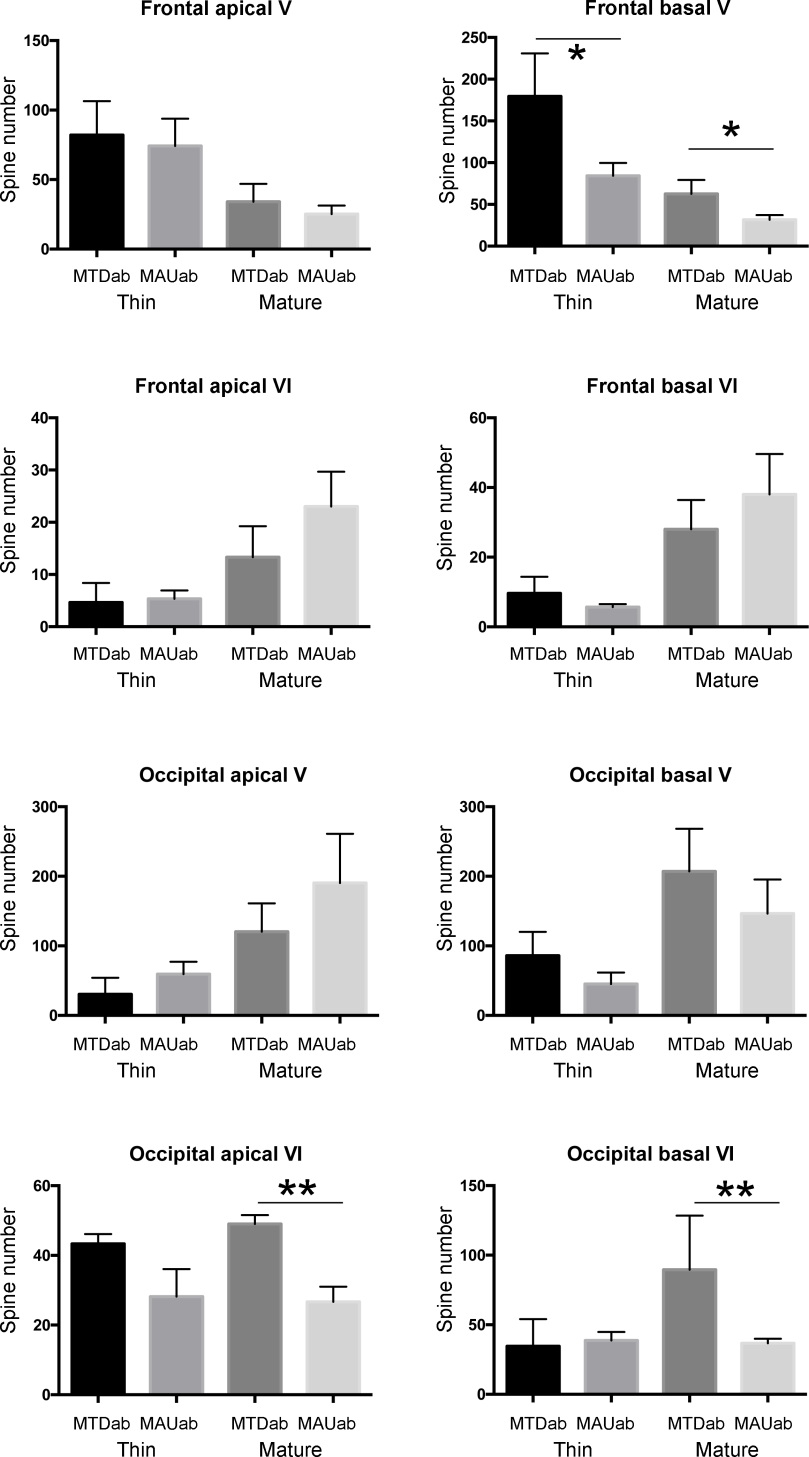
The total number of spines in basal dendrites of layer V pyramidal neurons was lower than in the control neurons. The total number of spines in apical and basal dendrites of layer VI pyramidal neurons in the occipital cortex was also decreased. The decrease in spine number in the occipital cortex was only due to a decrease in the number of mature spines. We did not find any change in other variables. Error bars (sem) are represented. Interestingly, the time of exposure to these antibodies (E14.5) coincided with the time of generation of pyramidal neurons in layer V in the frontal cortex and pyramidal neurons in layer VI in the occipital cortex, following the normal rostro-caudal pattern of cortical cell generation. One asterisk: p < 0.05; Two asterisks: p <0.01.

We detected an alteration in the layer V of the frontal cortex of MAU^ab^ offspring. We did not detect any changes in the remaining cortical layers (Figs 1, 2 and 3). The basal dendrites of layer V neurons were decreased in length (48% decreased, MAU^ab^, 51.01 ± 2.19, MTD^ab^: 97.45 ± 11.95, p = 0.04) and volume (67% decreased, MAU^ab^: 41.80 ± 5.0, MTD^ab^: 126.55.45 ± 28.07, p = 0.01), and both the total number of spines and the spine density were lower than in the control neurons. Basal dendrites in layer V in MAU^ab^ presented with a 57% decrease in the total number of spines (MTD^ab^: 279.5 ± 26.0, MAU^ab^: 120.66 ± 17.5, p = 0.001). Spine density was also lower in the MAU^ab^ group than in the control group (50% decreased, MTD^ab^: 2.25 ± 0.1, MAU^ab^: 1.14 ± 0.2, p = 0.005). Both the number of thin and mature spines were marginally decreased (thin spines decreased 64%, MTD^ab^: 179.5 ± 39, MAU^ab^: 84.3 ± 13, p = 0.06; mature spines decreased 50%, MTD^ab^: 62.5 ± 12, MAU^ab^: 31.5 ± 5 p = 0.07). We did not detect any modification in length or volume nor in the total number of spines in the frontal cortex in apical dendrites. To compensate for testing each individual hypothesis, we calculated the Bonferroni adjusted P value for statistical significance as P ≤ 0.002. When using this P value the decrease in the total number of spines in the basal dendrite was significant but not the spine density.

### Occipital cortex

We detected that in the occipital cortex of MAU^ab^ offspring there was an alteration in spines in layer VI of the cortex. We did not detect any changes in the rest of cortical layers (Figs 1, 2 and 3). We found a 37% decrease in the total number of spines in the MAU^ab^ group compared to the control group (MTD^ab^: 97.83 ± 3.1, MAU^ab^: 62.00 ± 12.7, p = 0.04), as well as a 32% reduction in the spine density in the apical dendrite (MTD^ab^: 0.25 ± 0.01, MAU^ab^: 0.17± 0.01, p = 0.005) of pyramidal neurons in the occipital cortex. This decrease in spine number was due to a decrease in the number of mature spines in the apical dendrite (46% decrease, 49.0 ± 2.4, MAU^ab^: 26.6 ± 4.1, p = 0.001). We also found a decrease in the number of mature spines in basal dendrites (60% decreased, MTD^ab^: 89.5 ± 30, MAU^ab^: 36.6 ± 3, p = 0.02). We did not note any other changes in the occipital cortex. When using the Bonferroni P value of significance, the decrease in the number of mature spines in the apical dendrite was significant but not in the basal dendrites.

Overall, intraventricular administration of MAU^ab^ during mouse fetal development at E14.5 did not affect dendrites or spines in the supragranular layers of the cortex, but we found a consistent decrease in the number of spines in infragranular layers in both cortical areas of interest, the frontal and occipital cortices. However, this alteration in spine number was only specific to layer V in frontal cortex and only to layer VI in occipital cortex. The number of mature spines and, to a lesser degree, the number of thin spines were affected. We also found a decrease in the length and volume of layer V basal dendrites in the frontal cortex of MAU^ab^ when compared to the MTD^ab^ treated mice.

## Discussion

We found that intraventricular administration of MAU^ab^ during mid stages of neurogenesis in mouse fetal development produced a consistent decrease in the number of spines in the infragranular layers in both cortical areas analyzed (frontal and occipital). The supragranular layers were not affected by the single autoantibody treatment. The prefrontal cortex is a region well-known to be affected in autism, while the occipital region has not yet been implicated. Nonetheless, in our experiments MAU^ab^ administration affected both cortices, indicating that MAU^ab^ interacted similarly with both cortical regions. However, this may not be the case in human, since the method of intraventricular injection we used allows for the antibodies to contact their target just for a limited time and bathes all the ventricular surface of the developing cortex. On the other hand, in humans there is a constant exposure to such antibodies and it is still not known if all the antibodies contact all the cortical areas equally. Nevertheless, this method allows us to evaluate what is the result of maternal autism-specific antibodies presence in the brain during development. We previously reported that MAU^ab^ administration during development affects neuronal soma size, and this increase affects neurons in all cortical regions and layers [16]. We also demonstrated that intraventricular exposure to autism-specific maternal autoantibodies produces autistic-like stereotypical behaviors in offspring mice [17].

Three main proteins have been discovered to be the ligands for the MAU^ab^. One of them is STIP1, which is first detected in the developing nervous system starting at E8 in the mouse [40]. STIP1 is a major ligand of the cellular prion protein (PrP(C)) and together they mediate neuritogenesis in cultured hippocampal neurons [41, 42]. Inhibition of this interaction leads to impaired memory formation in rodents [43], while a STIP1 50% reduction produces ASD-like phenotypes in mice, including hyperactive and attentional deficits [44]. STIP1 also modulates proliferation of astrocytes and retinal cells, and is associated with glioblastoma [45–47]. STIP1 is abundantly expressed in the cytoplasm and has been recently found to be associated with the actin cytoskeleton [48]. STIP1 directly interacts with the GTPase Rnd1, and overexpression of STIP1 prevents the Rnd1–plexin-A1-mediated cytoskeleton retraction. In PC-12 cells, overexpression of STIP1 enhances neurite outgrowth in the cellular processes initially established by Rnd1. It can be inferred from these studies that STIP1 participates in the Rnd1-induced signal transduction pathways that are involved in the dynamics of the actin cytoskeleton [49]. Our results demonstrating alteration in the development of the dendritic tree and the number of mature spines in the infragranular layers of the adult cortex after a brief exposure to MAU^ab^ are in accordance with the neuritogenic function of STIP1, one of the main targets of these antibodies.

Another major target of MAU^ab^ is the CRMP1 protein. The collapsin response mediator proteins (CRMPs 1–5) are thought to contribute to semaphorin-induced growth cone collapse [50], and are crucial for multiple neurodevelopmental processes. CRMP1 protein is highly expressed in the developing brain where it is required for cell migration as well [50, 51]. A third target of the MAU^ab^ is the mitochondrial enzyme lactate dehydrogenase (LDH), which is found in fetal brain [52] where it functions in cellular metabolism. Although autoantibodies targeting LDH have not yet been shown to play a direct role in altering neurodevelopment, the need for energy during brain growth and development make LDH an interesting target for MAU^ab^.

We reasoned that the changes we noted in the dendritic arbor and in the number of mature spines in infragranular layers of the cortex may be restricted to those layers because the time-limited exposure to MAU^ab^ during cortical development in our study. When administering antibodies in E14, we found dendritic and spine alterations in layer V in frontal cortex and in layer VI in the occipital cortex. Interestingly, the time of exposure to these antibodies coincides with the generation of pyramidal neurons in layer V in the frontal cortex and pyramidal neurons in layer VI in the occipital cortex, following the normal rostro-caudal pattern of cortical cell generation [53]. A reduction in the access of newborn cells to STIP1 in the developing cortex at the time of antibody administration may be responsible for the reduced dendritic arborization and reduced number of spines we noted in the adult cortex. We predict that in human, where MAU^ab^ are constantly present in the fetal brain during cortical development, the alterations described in pyramidal cells of our time-limited model may be present in all the layers of the cortex. Ideally, we will be following this study with an anatomical and pathology examination of a newly generated rodent model where the dam produces clinically relevant MAU^ab^ throughout pregnancy and lactation. Results from these ongoing studies will further aid in the determination of the pathologic mechanisms for MAU autism.
